# Locomotor activity: A distinctive index in morphine self-administration in rats

**DOI:** 10.1371/journal.pone.0174272

**Published:** 2017-04-05

**Authors:** Jian-Jun Zhang, Qingyao Kong

**Affiliations:** 1 CAS Key Laboratory of Mental Health, Institute of Psychology, Beijing, China; 2 Department of Psychology, University of Chinese Academy of Sciences, Beijing, China; 3 Laboratory of Neurobiology and the National Laboratory of Biomembrane and Membrane Biotechnology, College of Life Sciences, Peking University, Beijing, China; Scripps Research Institute, UNITED STATES

## Abstract

Self-administration of addictive drugs is a widely used tool for studying behavioral, neurobiological, and genetic factors in addiction. However, how locomotor activity is affected during self-administration of addictive drugs has not been extensively studied. In our present study, we tested the locomotor activity levels during acquisition, extinction and reinstatement of morphine self-administration in rats. We found that compared with saline self-administration (SA), rats that trained with morphine SA had higher locomotor activity. Rats that successfully acquired SA also showed higher locomotor activity than rats that failed in acquiring SA. Moreover, locomotor activity was correlated with the number of drug infusions but not with the number of inactive pokes. We also tested the locomotor activity in the extinction and the morphine-primed reinstatement session. Interestingly, we found that in the first extinction session, although the number of active pokes did not change, the locomotor activity was significantly lower than in the last acquisition session, and this decrease can be maintained for at least six days. Finally, morphine priming enhanced the locomotor activity during the reinstatement test, regardless of if the active pokes were significantly increased or not. Our results clearly suggest that locomotor activity, which may reflect the pharmacological effects of morphine, is different from drug seeking behavior and is a distinctive index in drug self-administration.

## Introduction

After Weeks [1] reported that rats voluntarily administered addictive drugs intravenously, self-administration (SA) of addictive drugs has been widely used as a tool for studying behavioral, neurobiological, and genetic factors in addiction for decades [2]. Thereafter, the progressive ratio break-point models [3,4] and the reinstatement model of relapse to drug-seeking behavior [5,6] were developed and extended current knowledge of incentive motivational factors in addiction and the mechanisms underlying relapse to drug SA. However, while most SA studies analyze and report active behavior (pokes or lever presses), inactive behavior and drug infusions, animals’ locomotor activity during SA sessions is not fully understood.

This is a relevant question, since hyperlocomotion is a critical index of the effects induced by drug abuse. The administration of abused drugs, for instance, methamphetamine, amphetamine, heroin, and ethanol, increases locomotor activity [7,8,9,10]. Morphine injected subcutaneously has previously shown remarkable locomotor-enhancing effects [11], which was considered to be one of the major side effects of morphine analgesia [12]. Moreover, the motor stimulant effects of opioids are closely related to the addictive properties of these drugs [12], and the locomotor enhancing action induced by morphine share similar receptor-regulated mechanisms with rewarding effects [12,13,14]. Similarly, researchers also found genetic correlations between effects of alcohol on locomotor activity and alcohol rewarding [15]. Finally, locomotor activity is commonly measured in behavioral sensitization tests, which has been proposed as a model for the development of drug dependence [16] and craving. This may be one of the underlying mechanisms responsible for high rates of relapse [17].

In the SA procedure, the instrumental behavior (pokes or lever press) of rodents may contribute to the measured locomotor activity. Therefore, to confirm whether locomotor activity is distinctive from drug seeking/taking behavior and can be an index in opioids SA, we generated morphine SA in rats and analyzed the locomotor activity during acquisition, extinction and reinstatement of morphine SA.

## Materials and methods

### Animals

Male Sprague-Dawley rats (220–250 g on arrival) were habituated to the animal facility for 1 week before behavioral experiments. Rats were housed under a reversed 12-h light/dark cycle with food and water *ad libitum* except for the morphine self-administration (SA) training [18], during which they received a 20-g daily ration of rat chow. All procedures were approved by the Institutional Animal Care and Use Committee of Peking University.

### Drugs

Morphine hydrochloride Injection (Shenyang First Pharmaceutical Factory, Shenyang, China) was diluted in sterile saline to obtain the dose of 1 mg/mL.

### Surgery

A silastic catheter (AniLab, Ningbo, China) was implanted in the right jugular vein and sutured in place under sodium pentobarbital (75 mg/kg, i.p.) anesthesia. After surgery, the catheter was flushed daily with 0.4 ml heparinized saline (100 IU/ml). Rats recovered for 7 to 10 days after surgery. Penicillin (160 000 IU/day) was administered in the first 4 days following surgery [19]. Rats experiencing catheter occlusion (n = 6) were excluded from the analysis.

### Morphine self-administration, extinction and relapse tests

#### Apparatus

The self-administration (SA) setup consisted of 16 operant chambers (AniLab, Ningbo, China). Each 8-beam infrared chamber (29 × 29 × 26 cm) was located in an opaque sound-proof box equipped with exhaust fans. Each chamber had a white house light for illumination. Two holes, placed at 5 cm from the grid floor, were used to record “nose poking”. A blue cue light was placed inside each hole. A speaker, located outside of the chamber, was used to provide audio cues. Daily, each rat was placed in an SA chamber and the catheter was connected to a pump-driven syringe (infusion speed, ~20 μl/sec). Locomotor activity was monitored and recorded as beam breaks throughout each SA session. Data were collected with PC Windows-compatible AniLab software (AniLab, Ningbo, China).

#### Acquisition

Rats were trained to self-administer morphine (0.3 mg/kg/100 μl infusion) in daily 3-h sessions on a fixed ratio 1 (FR1) schedule during their dark cycle [19,20]. The house light was on at the beginning of each session. Poking the nose into the active hole resulted in a 5-s morphine infusion simultaneously paired with a 5-s compound audiovisual cue, during which the house light was off. Poking the nose into the inactive hole had no scheduled consequences. The morphine infusion was followed by a time-out period of 15 s, during which nose pokes were ineffective and the house light was turned off.

All rats were trained for a minimum of 10 days and until they met a criterion of required infusions varying less than 15% over the last 3 consecutive training days [18,19,21]. Extinction procedures began the day after the rat met this criterion. Any rat that did not meet this criterion after 18 days of training was excluded. The procedures for saline SA training were identical to those of morphine SA training, except that rats underwent 14 training sessions and received saline instead of morphine.

#### Extinction

Extinction sessions were conducted for 3 h daily. During the extinction phase, the procedures were identical to those of training, except that morphine was not present.

Rats remained in extinction until extinct for a minimum of 7 days and both the active pokes and inactive pokes were less than 10 for 3 consecutive days [18,19]. Twenty-four hours after the last extinction session, the rats were reinstated with 5 mg/kg of morphine (i.p.). Rats which did not meet this criterion after 21 days of extinction were excluded.

#### Reinstatement

On the test day, rats were injected with morphine (5 mg/kg, i.p.) and immediately placed in the operant cages. The procedures of the reinstatement session were the same as the extinction session. Two groups of rats were identified: successful reinstatement group (active pokes in reinstatement minus active pokes in extinction ≥ 5) and failed reinstatement group (active pokes in reinstatement minus active pokes in extinction < 5).

### Data analysis

All the data were presented as mean ± S.E.M. Statistical tests were performed with GraphPad Prism 5 (GraphPad Software Inc., La Jolla, CA, USA) for the behavioral data and the correlation analysis. Differences were determined by a two-way or one-way repeated measures analysis of variance (ANOVA) followed by Bonferroni post hoc test. When comparing two groups, a Student’s t-test (two-tailed) was used. Pearson’s correlation analysis was used to assess the relationship between the self-administration behavior and the locomotor activity. * P < 0.05, ** P < 0.01 and *** P < 0.001 were considered as significant differences.

## Results

### Morphine self-administration enhanced the locomotor activity

**Fig 1:**
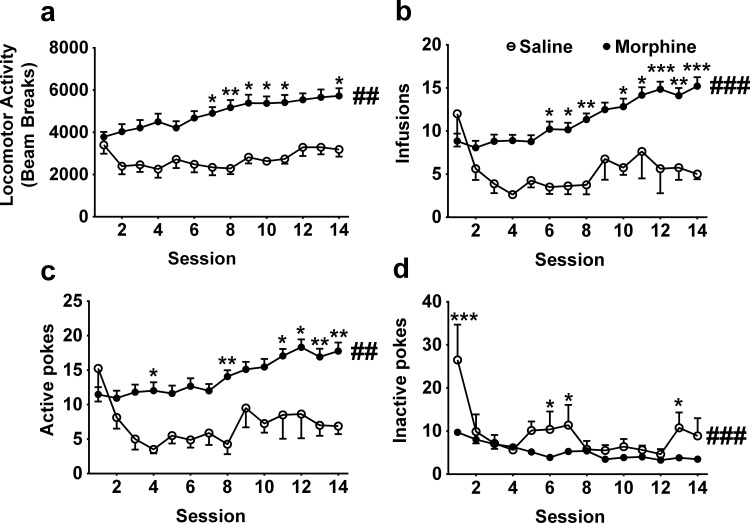
Locomotor activity during morphine self-administration. (a) The locomotor activity in the morphine SA group was higher than the saline SA group. (b) The infusions in the morphine SA group were higher than the saline SA group. (c) The active pokes in the morphine SA group were higher than the saline SA group. (d) The inactive pokes in the morphine SA group were lower than the saline SA group. ## p < 0.01, ### p < 0.001, compared to saline SA. * p < 0.05, ** p < 0.01,*** p < 0.001, post hoc test compared to saline SA on the same session. Saline SA, n = 8; morphine SA, n = 47.

To measure the locomotor response to self-administered morphine, we used two groups of rats: morphine SA training group and saline SA training group. For the 14 days of morphine or saline SA training (morphine: n = 47, saline: n = 8), a significant difference was found in the locomotor activity between the two groups (main effects of morphine: F _1, 53_ = 8.04, P <0.01; time: F _13, 689_ = 5.12, P <0.001; interaction: F _13, 689_ = 2.99, P <0.001; Fig 1A). One-way ANOVA confirmed that the locomotor activity progressively increased in the morphine SA group (F _13, 598_ = 19.47, P <0.001) but not in the saline SA group (F _13, 91_ = 1.75, P = 0.06). At the same time, higher infusions (main effects of morphine: F _1, 53_ = 13.88, P <0.001; time: F _13, 689_ = 5.73, P <0.001; interaction: F _13, 689_ = 4.53, P <0.001; Fig 1B), higher active pokes (main effects of morphine: F _1, 53_ = 11.36, P <0.01; time: F _13, 689_ = 4.04, P <0.001; interaction: F _13, 689_ = 3.34, P <0.001; Fig 1C), and lower inactive pokes (main effects of morphine: F _1, 53_ = 15.68, P <0.001; time: F _13, 689_ = 13.72, P <0.001; interaction: F _13, 689_ = 5.68, P <0.001; Fig 1D) were observed in the morphine SA training group compared with the saline SA training group. It is concluded that morphine SA effectively enhanced locomotor activity.

**Fig 2:**
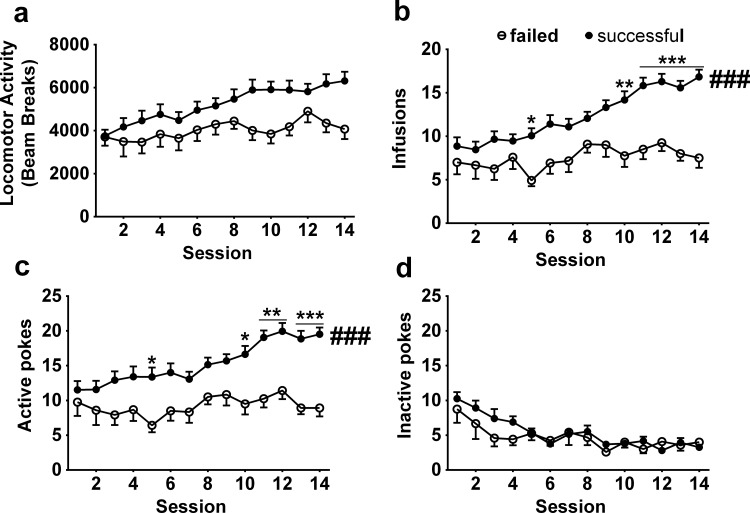
Locomotor activity in successful or failed acquisition of morphine SA. (a) The locomotor activity in the successful acquisition group was higher than in the failed acquisition group. (b) The morphine infusions in the successful acquisition group were higher than failed acquisition group. (c) The active pokes in the successful acquisition group were higher than failed acquisition group. (d) The inactive pokes had no significant difference between the successful acquisition group and failed acquisition group. ### p < 0.001, compared to failed acquisition group. * p < 0.05, ** p < 0.01,*** p < 0.001, post hoc test compared to failed acquisition group on the same session. Successful acquisition group, n = 35; failed acquisition group, n = 12.

Out of 47 rats, 35 successfully acquired morphine SA. The rats were grouped accordingly into a successful acquisition group and a failed acquisition group. We compared the locomotor response to morphine of these two groups, and marginally significant difference was found [main effects of group (successful/ failed): F _1, 45_ = 3.01, P = 0.09; time: F _13, 585_ = 11.17, P <0.001; interaction: F _13, 585_ = 3.54, P <0.001; Fig 2A]. To reduce the variance among different chambers and individuals, the locomotor activity was normalized to the percent change from the locomotor activity in the first training session. The normalized locomotor activity was analyzed and a significant difference was found (data not shown). Moreover, morphine infusions [main effects of group (successful/ failed): F _1, 45_ = 16.47, P <0.001; time: F _13, 585_ = 9.508, P <0.001; interaction: F _13, 585_ = 3.78, P <0.001; Fig 2B] and active pokes (main effects of group: F _1, 45_ = 16.78, P <0.001; time: F _13, 585_ = 5.16, P <0.001; interaction: F _13, 585_ = 2.39, P <0.01; Fig 2C) were higher in the successful acquisition group compared with the failed acquisition group. We then analyzed the inactive pokes in these two groups. The ANOVA confirmed significant main effects of time (F _13, 585_ = 7.23, P <0.001), but not of treatment (F _1, 45_ = 1.06; P = 0.31) or interaction effects (F _13, 585_ = 0.87; P = 0.59, Fig 2D). Thus, rats that successfully acquired morphine SA showed higher locomotor activity than the rats which failed to acquire SA. To evaluate the changes of locomotor activity, a one-way ANOVA confirmed that the locomotor activity progressively increased in the successfully acquired morphine SA group (F _13, 442_ = 23.64, P <0.001) but not in the rats that failed to acquire SA (F _13, 143_ = 1.82, P = 0.05).

### Locomotor activity level was correlated with morphine infusion numbers

**Fig 3:**
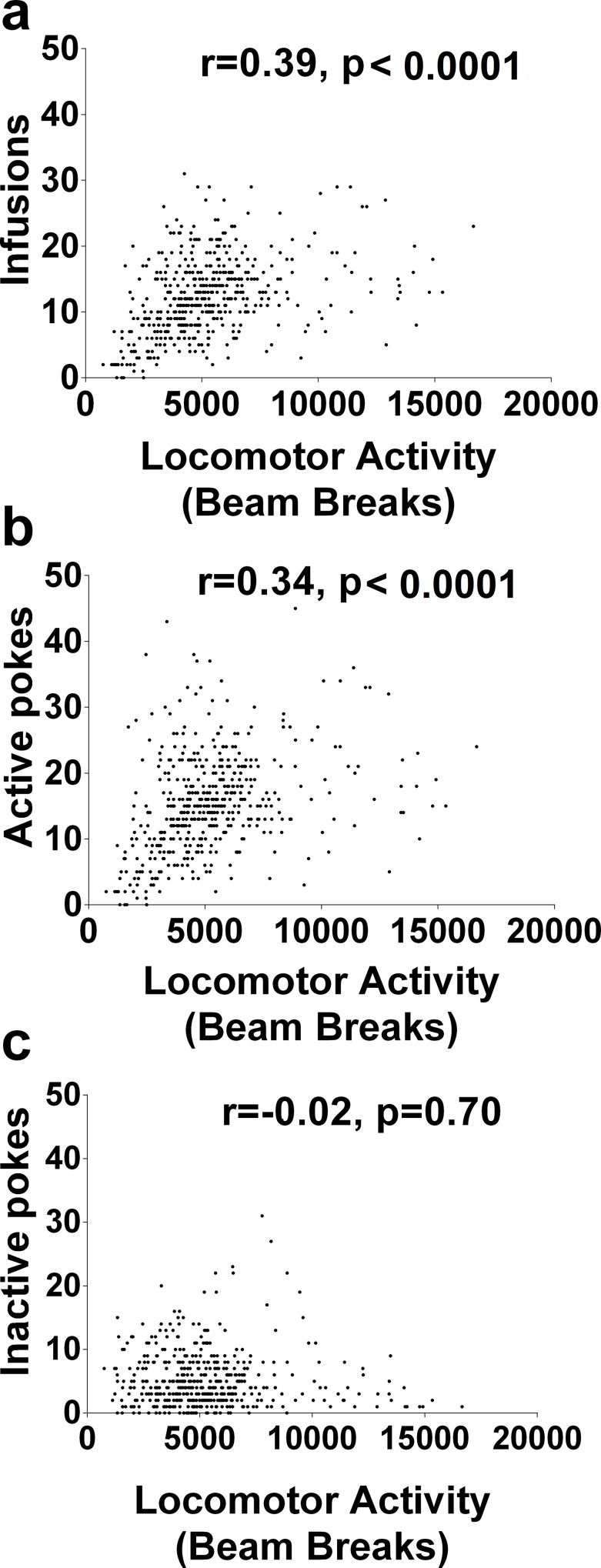
Locomotor activity was correlated with morphine infusion. (a) Locomotor activity in the successful acquisition group was correlated with morphine infusion. (b) Locomotor activity in the successful acquisition group was correlated with active pokes. (c) Locomotor activity in the successful acquisition group was not correlated with inactive pokes.

To explain why the locomotor activity increased in morphine SA training, we analyzed the correlation between locomotor activity and morphine infusion/pokes in the successfully acquired group of rats (n = 35). The positive correlation between locomotor response and morphine infusion was significant (r = 0.39, P <0.001, Fig 3A). The correlation between locomotor response and active pokes was also significant (r = 0.34, P <0.001, Fig 3B). On the contrary, as an indicator of novelty-exploring activity, inactive pokes had no significant correlation with locomotor response (r = -0.02, P = 0.70, Fig 3C). Thus, we assume that the main factor that contributes to increased locomotor activity is the pharmacological effects of morphine but not exploration more generally.

### The locomotor activity decreased in extinction

**Fig 4:**
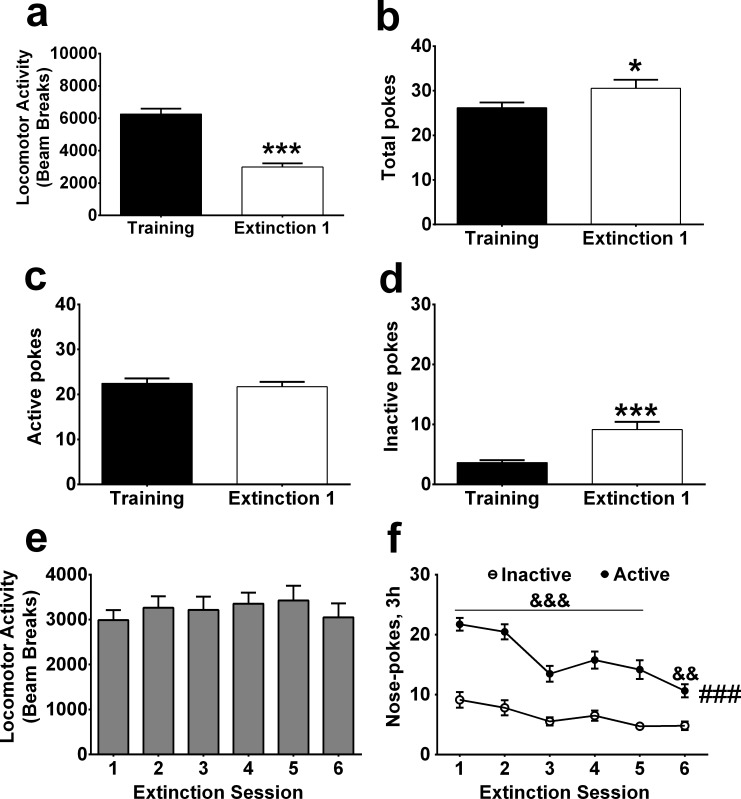
The locomotor activity decreased in the first extinction session. (a) Compared with the last acquisition, the locomotor activity decreased in the first extinction day (n = 47). (b) The active pokes did not significantly change between the first extinction day and the last acquisition day. (c) The inactive pokes significantly increased in the first extinction day compared with the last acquisition day. (d) The total pokes significantly increased in the first extinction day compared with the last acquisition day. (e) The locomotor activity did not change in the first six extinction days. (f) The active pokes were significantly higher than the inactive pokes in the first six extinction days. * p < 0.05, *** p < 0.001, compared to the last acquisition session. ### p < 0.001, compared to the inactive pokes. && p < 0.01, &&& p < 0.001, post hoc test compared to the inactive pokes on the same session.

After morphine SA was successfully acquired, extinction sessions were conducted for 3 h once daily (n = 47 in total, in which 35 rats were trained for at least 14 days, and the other 12 were trained for less than 14 days). We compared the locomotor activity and the number of pokes between the last acquisition session and the first extinction session. A paired t-test showed that locomotor activity significantly decreased in the first extinction session compared to the last acquisition session (t = 13.47, P <0.001, Fig 4A). Interestingly, the total pokes significantly increased in the first extinction session (t = 2.20, P <0.05, Fig 4B), while the active pokes had no significant difference between the two tests (t = 0.57, P = 0.57, Fig 4C) and the inactive pokes significantly increased in the first extinction session (t = 3.99, P <0.001, Fig 4D).

To investigate whether the decrease of locomotor activity can last for a long time, we analyzed the locomotor activity and nose-pokes in the first 6 consecutive extinction sessions. One-way ANOVA confirmed that the decrease of locomotor activity can last at least six days (F _5, 230_ = 1.34, P = 0.25, Fig 4E), although the active pokes are still significantly higher than the inactive pokes (main effects of group: F _1, 92_ = 97.55, P <0.001; time: F _5, 460_ = 9.51, P <0.001; interaction: F _5, 460_ = 3.63, P <0.01; Fig 4F).

From these results, we conclude that the high level of locomotor activity is maintained by the pharmacological effects of morphine but not by the drug-seeking or taking behavior.

### Morphine-primed reinstatement induced an increase in locomotor activity

**Fig 5:**
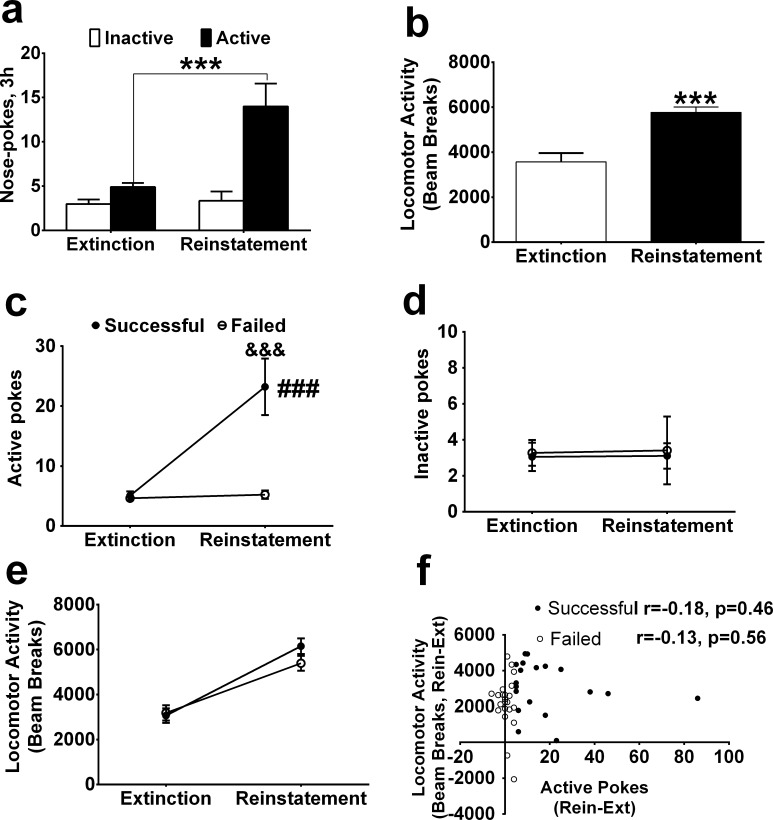
Morphine-primed reinstatement resulted in increase of locomotor activity. (a) Morphine induced reinstatement of drug-seeking behavior (n = 41). (b) Locomotor activity increased in the reinstatement test when compared to the last extinction day. (c) The active pokes in the successful reinstatement group (n = 20) were higher than in the failed reinstatement group on the reinstatement test (n = 21). (d) There was no difference in the inactive pokes between the successful reinstatement group and the failed instatement group. (e) Locomotor activity increased in the reinstatement test for both the successful reinstatement group and the failed reinstatement group. (f) The increase in locomotor activity was not correlated with the increase in active pokes in the successful reinstatement group or the failed reinstatement group. *** p < 0.001, compared to the last extinction session. ### p < 0.001, compared to the failed reinstatement group. &&& p < 0.001, post hoc test compared to the failed reinstatement group on the same session.

After morphine SA was fully extinguished, morphine-primed reinstatement was conducted (n = 41). For the active and inactive pokes, the ANOVA indicated significant main effects of pokes (F _1, 80_ = 18.15, P<0.001). There were significant effects of morphine treatment (F _1, 80_ = 11.53, P<0.01) and a significant interaction between morphine treatment and pokes (F _1, 80_ = 9.82; P<0.01, Fig 5A), this suggested that morphine successfully induced reinstatement. Meanwhile, for the locomotor activity, a t-test indicated that morphine-primed reinstatement had significantly higher locomotor activity compared to the last extinction session (t = 6.21, P<0.001, Fig 5B).

Two groups of rats were identified: the successful reinstatement group (active pokes (reinstatement) minus active pokes (extinction) ≥ 5, n = 19) and the failed reinstatement group (active pokes (reinstatement) minus active pokes (extinction) < 5, n = 22). For the active pokes, the ANOVA indicated significant main effects of group (successful/failed) (F _1, 39_ = 15.11; P<0.001), significant effects of morphine treatment (F _1, 39_ = 18.83; P<0.001), and a significant interaction across the last extinction and reinstatement (F _1, 39_ = 16.53; P<0.001, Fig 5C). A Bonferroni post hoc test showed that the successful reinstatement group had higher active pokes than the failed reinstatement group (t = 5.61, P <0.001). For the inactive pokes, the ANOVA indicated no significant effects of group (F _1, 39_ = 0.05; P = 0.82), morphine treatment (F _1, 39_ = 0.01; P = 0.94), or interaction (F _1, 39_ = 0.001; P = 0.97, Fig 5D) across the last extinction and reinstatement day. Moreover, locomotor activity increased in both successfully reinstated rats and failed reinstated rats, but no significant difference was found between these two groups (main effects: F _1, 39_ = 0.58, P = 0.45; effects of morphine treatment: F _1, 39_ = 137.1, P<0.001; interaction: F _1, 39_ = 3.77, P = 0.06, Fig 5E). Consistent with this finding, the positive correlation between locomotor response and active pokes was not significant (successful reinstated: r = -0.18, P = 0.46; failed reinstated: r = -0.13, P = 0.56, Fig 5F). These results indicated that regardless of if morphine induced remarked reinstatement or not, morphine can induce an increase in locomotor activity.

## Discussion

Our study examined the locomotor activity during the acquisition, extinction and reinstatement of morphine self-administration. Morphine SA enhanced locomotor activity compared to a saline control, and rats that successfully acquired SA had higher locomotor activity than those that failed. Moreover, locomotor activity was not related to baseline exploratory behavior, but did correlate with the amount of drug taken. More interestingly, the drug-free state in extinction procedures resulted in a lack of morphine-stimulated locomotion, while morphine priming induced hyperlocomotion in the reinstatement test. Our results may extend the drug abuse research field using SA model by isolating the locomotor activity from the proactive behavior in drug addiction, and by showing an involvement of locomotor activity in pharmacological effects of morphine itself.

Our results showed that compared with saline SA, morphine SA rats had higher locomotor activity. Although a previous study showed behavioral sensitization effects in heroin SA training [22], we found that the locomotor activity progressively increased during the acquisition phase, which is not behavioral sensitization because of the increased morphine self-administered. The differences between these two findings could occur due to differences in drug and experimental protocol. Moreover, we found that rats which successfully acquired morphine SA showed higher locomotor activity than the rats that failed. The hyperlocomotion may be due to increased drug-taking related behavior (e.g. active and inactive pokes) and/or locomotor behaviors in response to morphine intake.

Our results showed that both the positive correlation between locomotor response and morphine infusion, and the correlation between locomotor activity and active pokes, were significant. However, since a FR1 schedule was used and morphine infusion was tightly correlated with active pokes, these correlations could not demonstrate whether morphine itself or the operant behavior induce the increased locomotor activity. Considering that inactive pokes were always used as a measure of nonspecific activity and/or response generalization [18,23], we analyzed the correlation between locomotor response and inactive pokes. The correlation between these two factors was not significant, which suggested that the nonspecific activity didn’t increase and only the drug-related activity increased. More powerful evidence was that the locomotor activity decreased on the first extinction test, but the active pokes did not change; the total pokes even increased for the increased inactive pokes. Moreover, the decreased locomotor activity was maintained for at least six days although the active pokes were still significantly higher than the inactive pokes. This is an interesting result, suggesting that the increased locomotor activity is due to the pharmacological effects of morphine itself, but not because of the increased response generalization or the drug-seeking/taking behavior.

Our results, which showed that the locomotor activity increased after morphine primed regardless of whether the reinstatement was successful or not, afforded another solid evidence for the above postulation. Moreover, these results were also helpful in understanding the controversial relationship between enhanced locomotor activity and drug-primed reinstatement. One study found that enhanced locomotion was associated with heroin-primed reinstatement of active responding [18], but another study found extended access to cocaine SA enhances drug-primed reinstatement but not behavioral locomotor stimulation [24]. Our results strongly supported the second one and suggested that different mechanisms may be involved in the reinstatement of drug taking behavior and drug-primed hyperlocomotion.

The morphine dosage used in the present study, which was consistent with our previous studies [19,25], is a low dose. The locomotor responses to different doses of morphine are still unknown. Moreover, we have not tested the possible neural circuit or molecular mechanisms underlying the distinctive effects of locomotor activity and drug seeking behavior during the SA model. Thus, a series of questions need to be addressed in the future.

## Conclusions

In conclusion, our study shows, for the first time, that locomotor activity changes in the different phases of drug SA. Locomotor activity increases when morphine is administered and decreases when morphine is withdrawn. These findings strongly suggest that locomotor activity is a distinctive sign in SA model, and studies designed to measure locomotor activity and addictive behaviors during the same SA session in the same animals may afford a better model to compare the mechanisms underlying drug induced hyperlocomotion and drug seeking/taking behavior.

## Supporting information

S1 Dataset Data Underlying the Findings Presented in this Article. (XLSX)
